# Mitochondrial Morphogenesis, Dendrite Development, and Synapse Formation in Cerebellum Require both Bcl-w and the Glutamate Receptor δ2

**DOI:** 10.1371/journal.pgen.1000097

**Published:** 2008-06-13

**Authors:** Qiong A. Liu, Helen Shio

**Affiliations:** 1 Medical Department, Brookhaven National Laboratory, Upton, New York, United States of America; 2 Bio-Imaging Resource Center, Rockefeller University, New York, New York, United States of America; University of Minnesota, United States of America

## Abstract

Bcl-w belongs to the prosurvival group of the Bcl-2 family, while the glutamate receptor δ2 (Grid2) is an excitatory receptor that is specifically expressed in Purkinje cells, and required for Purkinje cell synapse formation. A recently published result as well as our own findings have shown that Bcl-w can physically interact with an autophagy protein, Beclin1, which in turn has been shown previously to form a protein complex with the intracellular domain of Grid2 and an adaptor protein, nPIST. This suggests that Bcl-w and Grid2 might interact genetically to regulate mitochondria, autophagy, and neuronal function. In this study, we investigated this genetic interaction of Bcl-w and Grid2 through analysis of single and double mutant mice of these two proteins using a combination of histological and behavior tests. It was found that Bcl-w does not control the cell number in mouse brain, but promotes what is likely to be the mitochondrial fission in Purkinje cell dendrites, and is required for synapse formation and motor learning in cerebellum, and that Grid2 has similar phenotypes. Mice carrying the double mutations of these two genes had synergistic effects including extremely long mitochondria in Purkinje cell dendrites, and strongly aberrant Purkinje cell dendrites, spines, and synapses, and severely ataxic behavior. Bcl-w and Grid2 mutations were not found to influence the basal autophagy that is required for Purkinje cell survival, thus resulting in these phenotypes. Our results demonstrate that Bcl-w and Grid2 are two critical proteins acting in distinct pathways to regulate mitochondrial morphogenesis and control Purkinje cell dendrite development and synapse formation. We propose that the mitochondrial fission occurring during neuronal growth might be critically important for dendrite development and synapse formation, and that it can be regulated coordinately by multiple pathways including Bcl-2 and glutamate receptor family members.

## Author Summary

A neuron cell is composed of cell body, axons, and dendrites. Dendritic spines on dendrites form synapses with axons of other neurons, establishing communication between neuron cells. Dendrite development and synapse formation are therefore important for neuronal function. Although many genes have been previously identified as affecting the development of dendrites and synapses, the apoptosis Bcl-2 family members have not yet been shown to regulate these processes. In this study, a Bcl-2 family survival member, Bcl-w, was found not to affect cell death, but to be required for synapse formation and motor learning in mouse cerebellum. Bcl-w also appears to control dendrite development as double null mutant mice of Bcl-w and the glutamate receptor δ2 (Grid2) have severe defects in Purkinje cell dendrites, spines, and synapses. In addition, Bcl-w and Grid2 act synergistically to promote what is likely to be mitochondrial fission in Purkinje cells. Neither the survival members of the Bcl-2 family nor the excitatory receptors have been demonstrated previously to regulate mitochondrial morphogenesis in brain. We conclude that neuronal dendrite development and synapse formation require perhaps mitochondrial fission that can be controlled by two critical pathways including Bcl-w and Grid2.

## Introduction

Mitochondria have been shown to undergo morphological changes in many neurodegenerative and psychiatric diseases, suggesting their vital role in maintaining the normal function of neuron cells. One of the morphological changes in mitochondria is the length or size, which can be controlled by mitochondrial growth or mitochondrial fission/fusion cycles. Mitochondria are dynamic organelles that can undergo fission, fusion, branching, and change in subcellular distribution [1]–[3], resulting in the exchange of their genetic materials, alteration of their shape, and increase or decrease of their number [1]–[3]. This dynamic nature of mitochondria is also critically important for energy generation, calcium buffering, and control of apoptosis. Mitochondrial fission and fusion is normally a well-balanced event; when the fission is blocked, the length of mitochondria increases due to ongoing fusion, and mitochondrial fission sites persist as constriction sites due to the slowdown of fission, whiles when the fusion is inhibited, mitochondria usually appear fragmented [3]. Mitochondrial number increases during cellular division, growth, and differentiation via the fission process [4]. However, excessive fission can stimulate apoptosis [5], and cause neurodegenerative diseases [6]. In cultured healthy neurons, mitochondrial fission and fusion proteins have been shown to regulate the morphology and plasticity of dendritic spines and synapses [7]. In addition, glutamate [8] and synaptic activity [7] modulates the motility and fusion/fission balance of mitochondria and controls mitochondrial distribution in dendrites [7]. Several proteins have been identified in a variety of species to mediate mitochondrial fission or fusion process [2],[3], however, little is known about the signaling molecules that activate these processes.

Cerebellar Purkinje cells are characterized by large and highly branched dendritic arbors in the brain. Over 90% of Purkinje cell dendritic spines form excitatory synapses with granule cell parallel fiber axons, which relay information from pre-cerebellar nuclei to Purkinje cells. Grid2 is strongly expressed in Purkinje cells [9], and localizes specifically to Purkinje cell/ parallel fiber synapses [10],[11]. Analysis of Grid2 knockout mice [12], and *Hotfoot* mice carrying spontaneous loss-of-function mutations in Grid2 [13],[14] has demonstrated that these mice exhibit an impaired function on motor coordination and learning tasks, and have structural and functional defects in Purkinje cell/granule cell parallel fiber synapses and altered long term depression [12],[15],[16]. Physiologic studies of Grid2^Lc^, the Lurcher dominant mutation have established that the Grid2^Lc^ mutation results in inward Ca^2+^/Na^+^ current and constitutive activation of the δ2 glutamate receptor, and also that the Grid2^Lc^ receptor has similar channel properties to both NMDA [17] and AMPA receptors [18],[19]. Activation of Grid2^Lc^ also induces autophagy and degeneration of Purkinje cells. This degeneration might be mediated through interaction of Grid2 with its downstream autophagy protein, Beclin1 [20]. Autophagy is a conserved mechanism for degradation of proteins and other subcellular constituents, and is often involved in cell and tissue remodeling or cell death [21]. Two recent reports demonstrated that Purkinje cells also degenerate without the presence of the basal level of autophagy [22],[23].

Bcl-2 family members have been most extensively studied in the context of apoptotic cell death [24]. The Bcl-2 family was divided into the pro-survival members that protect cells from being killed, and the pro-death members that kill cells. Bcl-w belongs to the pro-survival group of the Bcl-2 family that includes Bcl-2, Bcl-XL, A1, and CED-9 [25],[26]. These proteins function to protect cells from apoptosis by binding to the outer membrane of mitochondria through their C-terminal hydrophobic domain, thereby preventing the release of several apoptosis proteins from mitochondria into the cytoplasm. They include the caspase regulatory proteins and proteins that lead to DNA fragmentation and chromosome condensation [27]. Bcl-w is widely expressed in a variety of tissues, but predominantly in adult brain and spinal cord [28]. The expression of Bcl-w in brain increases during the postnatal development and is maintained at high levels in the adult brain including cerebellum Purkinje cells, where it is localized to Purkinje cell soma [29] and dendrites (Lab Vision Corporation), whereas Bcl-XL, the only other pro-survival member that is expressed in adult brain had much lower level of expression [29]. *Bcl-w^−/−^* mice are smaller during the early postnatal development, but viable and normal in appearance as adults. Both apoptotic and non-apoptotic cell death have been observed in the testes of *Bcl-w^−/−^* mice [30],[31].

A recent report as well as our own findings demonstrated that several other survival members of the Bcl-2 family including Bcl-w could also bind to the autophagy protein, Beclin1 [32]–[34]. Beclin1 has been shown previously to form a protein complex with an adaptor protein, nPIST, and the intracellular domain of Grid2 [20]. Thus, Bcl-w might interact genetically with Grid2 to regulate mitochondrial, autophagy, and neuronal function.

In this study, we aim to understand how Bcl-w and Grid2 interact genetically to regulate mitochondria, autophagy, and neuronal function using Bcl-w and Grid2 null mutant mice. We show that the survival member of the Bcl-2 family member, Bcl-w does not control the cell number in brain, but promotes what is likely to be the mitochondrial fission in Purkinje cell dendrites, and is required for the Purkinje cells/parallel fibers synapses and motor learning. We demonstrated that the excitatory receptor Grid2 could regulate mitochondrial length, and the mutation of this protein shares the similar phenotypes in cerebella with the loss-of-function of Bcl-w. Comparative analyses of single and double mutant mice of Bcl-w and Grid2 further indicate that these molecules act synergistically to regulate mitochondrial length and to control the development of Purkinje cell dendrites, dendritic spines, and synapse formation. We further show that no evidence of alteration of autophagy in single and double mutant mice was observed, and the potential upregulation of Beclin1 in *Bcl-w^−/−^* mice and overexpression of Beclin1 was not sufficient to activate autophagy. We have thus identified Bcl-w and Grid2 as two critical proteins acting in distinct pathways to control mitochondrial morphogenesis and Purkinje cell development in the mouse cerebellum.

## Results

### Bcl-w and Grid2 Activity Regulate Mitochondrial Length in Purkinje Cell Dendrites

Since Bcl-w binds to Beclin1, which in turn can form a protein complex with nPIST and the intracellular domain of Grid2 [20], the possibility arises that Bcl-w may function downstream of Grid2. We thus examined if *Bcl-w^−/−^* [30],[31] and *Grid2^ho−4J(−/−)^* mice [14],[35],[36] that carry spontaneous null mutation of the Grid2 gene share similar phenotypes. Since Bcl-w binds to the outer membrane of mitochondria to regulate apoptotic activity [26], we examined both cell numbers (see below) and the morphology of mitochondria in *Bcl-w^−/−^* and *Grid2^ho−4J(−/−)^* mice by electron microscopy (EM). Purkinje cells were focused on because Grid2 is only expressed in Purkinje cells [9]–[11], and Bcl-w also had strong expression in these cells in adult brain [28],[29].

**Figure 1:**
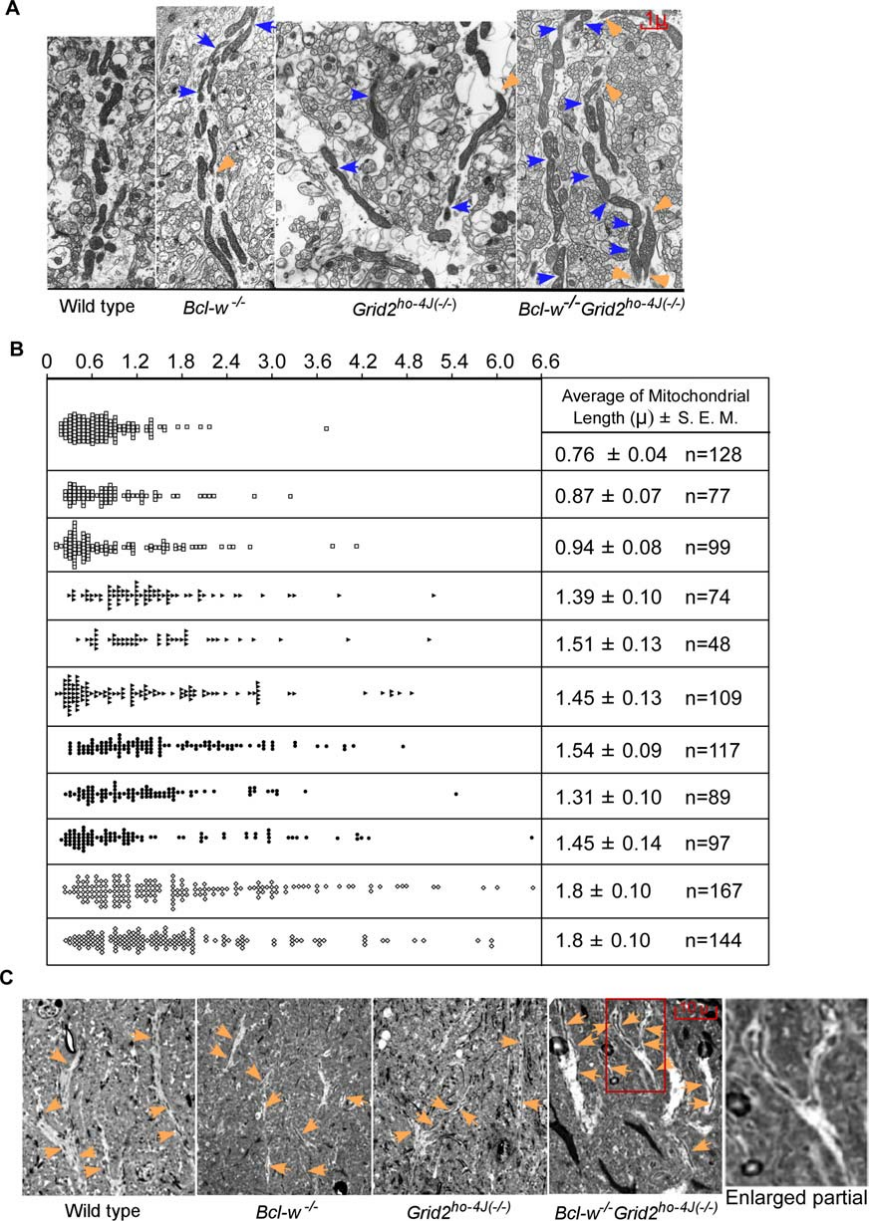
Mitochondrial Morphology Abnormality in Purkinje Cell Dendrites of *Bcl-w^−/−^*, *Grid2^−/−^,* and *Bcl-w^−/−^Grid2^ ho−4J/(−/−)^* Mice. (A) Electronmicrograph to show the morphology of mitochondria in dendritic track in wild type, *Bcl-w^−/−^*, *Grid2^−/−^,* and *Bcl-w^−/−^Grid2^ ho−4J/(−/−)^* mice. Black arrows indicate dendritic spines, orange arrowhead the broken end of mitochondria, indicating mitochondria transiting out of section plate, and the blue arrows the constriction sites on mitochondria. (B) Histograph showing the length of mitochondria in Purkinje cell dendrites. Each lane represents one mouse. Square indicates wild type mice, solid triangle the *Bcl-w^−/−^* mice, solid dot the *Grid2^−/−^*mice, and diamond the *Bcl-w^−/−^Grid2^ ho−4J(−/−)^* mice. A student t-test was performed on mitochondrial length among all listed mice. Three wild type mice are significantly different from all other mice in mitochondria length, p<0.0001. The mitochondrial lengths in two *Bcl-w^−/−^Grid2^ho−4J(−/−)^* mice are significantly different from that in single knockout mice, p<0.05. “n” indicates the number of mitochondria collected. (C) Semi-thin sections illustrating the presence of mitochondria in dendritic tracks in wild type, *Bcl-w^−/−^*, *Grid2^−/−^,* and *Bcl-w^−/−^Grid2^ ho−4J(−/−)^* mice. The orange arrows point at mitochondria in Purkinje cell dendrites.

In these EM micrograph, profiles of mitochondria collected from longitudinal sections of dendritic tracks in electron micrographs appear lengthened in both *Bcl-w^−/−^* and *Grid2^ho−4J(−/−)^* mice (Figure 1A). The lengths of mitochondria were thus measured and quantified. Palay and Chan-Palay [37] have demonstrated using EM method that the mitochondrial lengths in Purkinje cells of wild type mice are ∼0.1–0.6 μ. In the present study, wild type mice yielded an average value ∼0.7 – 0.8 μ, with about two third of mitochondria measuring between 0.1–0.8 μ (Figure 1B). This is similar to the previous electron micrographic estimates [37]. In *Bcl-w^−/−^* mice, the average length of mitochondria was increased to ∼1.4–1.5 μ (Figure 1A, B), similarly to that in *Grid2^ho−4J(−/−)^* mice, ∼1.3–1.6 μ. Both numbers are significantly different from that obtained in wild type mice (Figure 1B). In addition, it was notified upon detailed examination of the micrographs that mitochondria in both *Bcl-w^−/−^* and *Grid2^ho−4J(−/−)^* mice often contained points where they became constricted (Figure 1A). This observation suggests that the lengthened mitochondria might be due to the inhibition or slowdown of mitochondrial fission process.

### Bcl-w and Grid2 Act Synergistically to Regulate Mitochondrial Length in Purkinje Cells

To understand if Bcl-w and Grid2 act in the same or separate pathways, we generate double mutant mice of Bcl-w and Grid2. The reason for this was that if mitochondrial and synaptic phenotypes in *Bcl-w^−/−^ Grid2^ ho−4J(−/−)^* double null mutant mice was similar to that in either of the single knockouts, then it can be concluded that Grid2 and Bcl-w function in the same pathway; whereas a finding be concluded that the additive or synergistic phenotypes of Bcl-w and Grid2 mutants would suggest that they act instead in distinct pathways. EM analysis of mitochondria in Purkinje cell dendrites of *Bcl-w^−/−^Grid2^ ho−4J(−/−)^* mice indicated that the average value ∼1.8–1.9 μ (Figure 1A, B) was significantly longer than those mitochondrial profiles obtained from the single mutant mice (Figure 1A, B). In addition, mitochondrial profiles from *Bcl-w^−/−^Grid2 ^ho−4J(−/−)^* mice contained frequent thinning and constriction sites (Figure 1A; blue arrows), and in some cases their cristae appeared slightly dilated (Figure 1A), indicating perhaps much slower mitochondrial fission compared to single and wild type mice.

The mitochondrial length estimated in *Bcl-w^−/−^Grid2^ ho−4J(−/−)^* mice is likely to be much underestimated length because very long mitochondria transit out of plate on very thin EM sections. In order to view the morphology of large mitochondria in Purkinje cells, we thus made thicker, 0.5 μ semi-thin plastic sections in distal Purkinje cell dendrites of wild type, single, and double mutant mice (Figure 1C). Intriguingly, many extremely long mitochondria (often >10 μ), which can extend for much of the visible length of Purkinje cell dendrites were frequently found in *Bcl-w^−/−^ Grid2^ ho−4J(−/−)^* double mutant mice (Figure 1C). In addition, small mitochondria in dendrites that contain extremely long mitochondria seem depleted. However, these were rarely found in single, and not at all in wild type mice. Since the mitochondrial length in double mutant mice seem longer than the sum of that in the single mutant mice in semi-thin sections, this supports strongly that Bcl-w and Grid2 genes interact synergistically rather than additively to control the mitochondrial length, and that the severely increased mitochondrial length in *Bcl-w^−/−^ Grid2^ ho−4J(−/−)^* double mutant mice might result from significant slowdown of the mitochondrial fission in Purkinje cell dendrites.

### Both Bcl-w and Grid2 Regulate the Mitochondrial Length in Non-Degenerating Purkinje Cells

**Figure 2:**
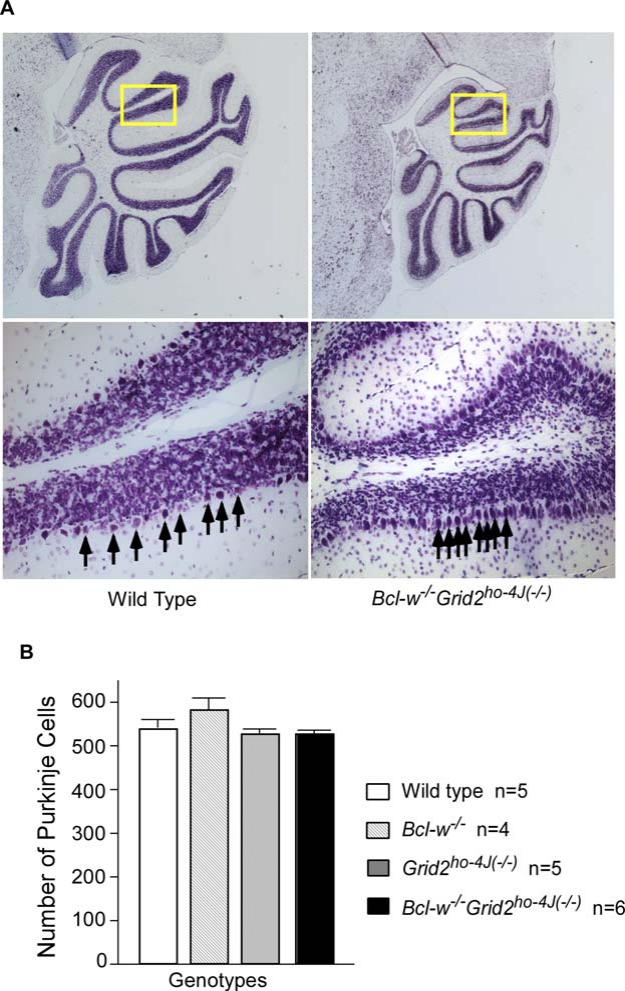
No Loss of Purkinje Cells in *Bcl-w^−/−^, Grid2^ ho−4J(−/−)^* or *Bcl-w^−/−^Grid2^ ho−4J(−/−)^* Double Mutant Mice. (A) Nissil stain showing the smaller cerebellum (top panel) and overcrowded Purkinje cell layer (bottom panel) in a *Bcl-w^−/−^Grid2^ ho−4J(−/−)^* double mutant mouse (right panels) relative to wild type mouse (left panels). (B) Bar graph demonstrating the average Purkinje cell numbers obtained from wild type, *Bcl-w^−/−^, Grid2^ ho−4J(−/−)^*, and *Bcl-w^−/−^Grid2^ ho−4J(−/−)^* mutant mice. Statistical analysis indicated no significant difference among Purkinje cell numbers of these genotypes. Error bars represent standard error of the mean.

We next examined if Purkinje cell number was altered in *Bcl-w^−/−^*, *Grid2^ho−4J(−/−)^* and *Bcl-w^−/−^Grid2^ ho−4J(−/−)^* mice. The rational for this experiment is that cell death has been observed in testes of Bcl-w knockout mice [30],[31] and in Purkinje cells of Grid2^Lc^ mutant mice [17], and that mitochondrial fission can stimulate apoptosis and cell degeneration. In these studies, we found that *Bcl-w^−/−^* mice brains appeared grossly normal, and no significant difference in Purkinje cell number (Figure 2A, B), or obvious changes in neuron number in other brain regions compared to wild type mice were observed (data not shown). A similar result was obtained in *Grid2^ho−4J(−/−)^* mice, agreeing with previous studies of Grid2 mutant mice [38]. Despite the fact that cerebella from the *Bcl-w^−/−^ Grid2^ ho−4J(−/−)^* animals were obviously smaller and contained an overcrowded Purkinje cell monolayer (Figure 2A), normal Purkinje cell numbers were found in the double mutant mice (Figure 2B). This result suggests that Bcl-w and Grid2 promote perhaps mitochondrial fission in non-degenerating Purkinje cells.

### Bcl-w Is Required for Synapse Formation and Motor Learning, Similarly to Grid2

**Figure 3:**
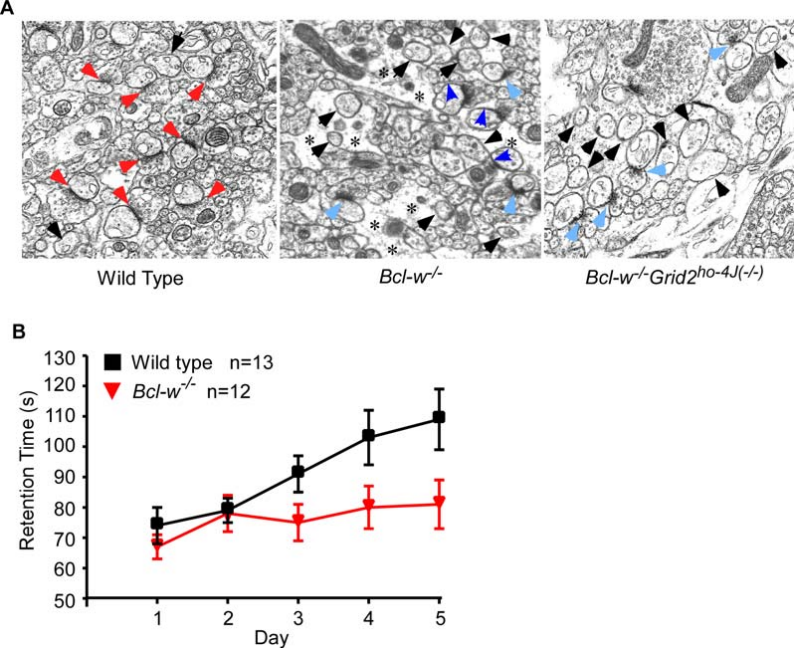
Abnormal Synapses and Rotarod Defect in *Bcl-w^−/−^* Mice. (A) Electromicrographs of synapses in wild type, *Bcl-w^−/−^*, and *Bcl-w^−/−^Grid2^ho−4J(−/−)^* mice. Red arrows indicate the normal synapses, light blue arrows the defective synapses with thick postsynaptic density, dark blue arrowheads the synapses with mismatched pre- and post- synaptic elements, black arrows the naked spines, asterisks the swelling Bergmann Glia encompassing Purkinje cells and synapses. (B) The learning profiles as represented by the average retention time for mice to stay on accelerating Rotarod bar during five consecutive testing days. The *Bcl-w^−/−^* mice had significant difference in retention time from that of wild type mice in day 2, 3, 4, and 5, P<0.001.

**Figure 4:**
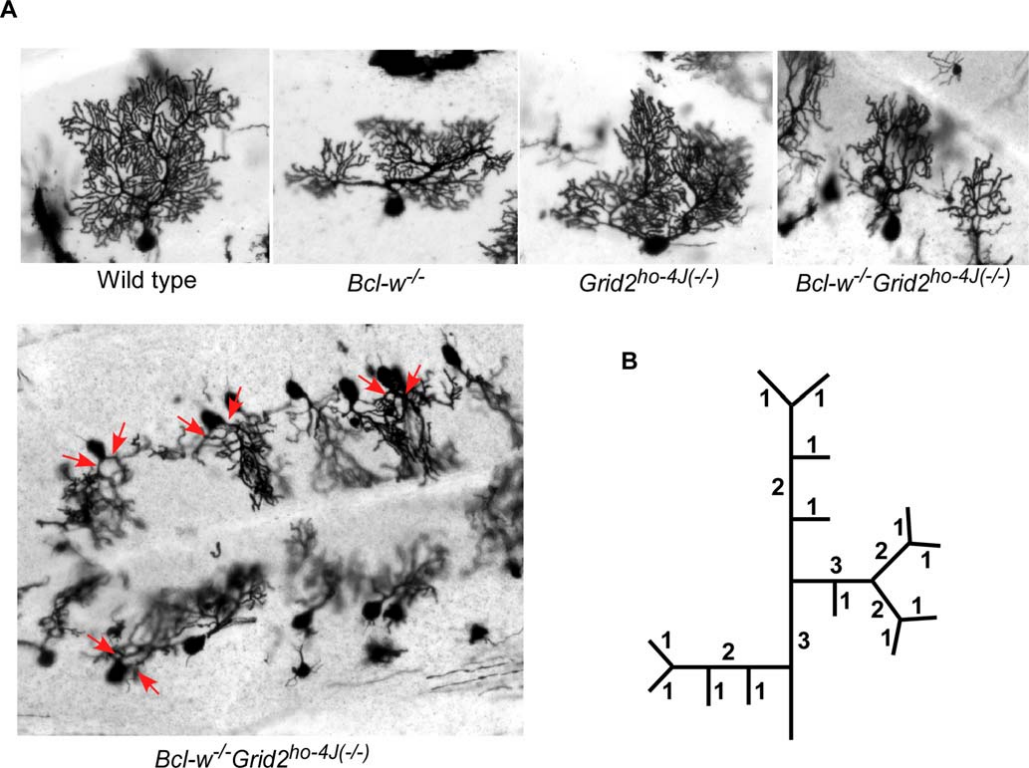
Golgi Stains Illustrating Dendritic Abnormalities in *Bcl-w^−/−^Grid2^ ho−4J(−/−)^* Double Mutant Mice. (A) Golgi stain showing the profile of Purkinje cell dendritic trees in wild type, *Bcl-w^−/−^*, *Grid2^ho−4J(−/−)^,* and *Bcl-w^−/−^Grid2^ho−4J(−/−)^* mice (top panel). Low magnification view of one partial folia in the *Bcl-w^−/−^Grid2^ ho−4J(−/−)^* cerebellum illustrating several affected Purkinje cells (lower panel). Red arrows indicate two primary dendritic branches extended from the Purkinje cell soma. (B) The Strahler order of method. This method was designed to assess topological features of dendrites by assigning a relative order of magnitude to each dendritic branch. The number of Strahler orders, combined with the number of branches in each of these orders, is a quantitative measure of the complexity of a dendritic tree. The tip branches are counted as “order 1”, and two “order 1” branches meet to form the “order 2” branch, etc.

Previous EM studies of Grid2 null mutant mice have revealed a large number of naked Purkinje cell dendritic spines, and mismatched connections between the pre- and postsynaptic active zones of Purkinje cell/parallel fiber synapses [12],[35]. Interestingly, our EM analysis of these synapses in *Bcl-w^−/−^* mice also indicated a large number (∼44% of total spines) of naked spines, and several synaptic defects including mismatched connections, shortened active zones, and thickened postsynaptic densities (Figure 3A; Table 1). Although the average number of naked spines did not change between *Grid2^ho−4J(−/−)^* (∼50% –70%) and *Bcl-w^−/−^Grid2^ho−4J(−/−)^* mutant animals (∼65%) (Table 1), there were much fewer synapses in the double mutant mice due to the significantly reduced number of dendritic arbors (see below; Figure 4).

**Table 1:** Percentage of Naked Purkinje Cell Spines in Total Spines in Wild Type, *Bcl-w^−/−^*, *Grid2^ho−4J(−/−)^*, *and Bcl-w^−/−^Grid2^ho−4J(−/−)^* Mice.

Genotypes	Naked Spines	Total Spines (Naked spines+Synapses)	Percentage of the Naked Spines (%)	Percentage of the Naked Spines (%)±S. E. M.
Wild type	54	224	24	24.7±1.2
Wild type	77	288	27	
Wild type	60	263	23	
*Bcl-w^−/−^*	107	282	38	44.3±5.4
*Bcl-w^−/−^*	130	238	55	
*Bcl-w^−/−^*	111	280	40	
*Grid2^ho−4J(−/−)^*	91	170	54	63.5±9.5
*Grid2^ho−4J(−/−)^*	267	365	73	
*Bcl-w^−/−^Grid2^ho−4J(−/−)^*	225	340	66	65.3±1.8
*Bcl-w^−/−^Grid2^ho−4J(−/−)^*	211	342	62	
*Bcl-w^−/−^Grid2^ho−4J(−/−)^*	179	263	68	

Quantitation of naked spines and synapses collected from 633–1392 um^2^ of molecular layer one third down from pia. The percentage of naked spines in wild type mice is significantly different from that in *Bcl-w^−/−^* with p<0.05, *Grid2^ho−4J(−/−)^* with p<0.01, *Bcl-w^−/−^Grid2^ho−4J(−/−)^* mice with p<0.01, the percentage of naked spines in *Bcl-w^−/−^* is significantly different from that in *Bcl-w^−/−^Grid2^ho−4J(−/−)^* mice with p<0.01, and there is no significant difference between *Bcl-w^−/−^* and *Grid2^ho−4J(−/−)^*, *Grid2^ho−4J(−/−)^* and *Bcl-w^−/−^Grid2^ho−4J(−/−)^* mice.

Using the accelerating Rotarod behavioral test [12],[15], we obtained evidence that the synaptic defects in the *Bcl-w^−/−^* mice observed in EM studies may result in deficits in cerebellar motor learning function in these mice (Figure 3B). We found that whereas wild type mice improved their performance with experience on the rotating bar, *Bcl-w^−/−^* mice consistently failed to improve in performance throughout the trials (Figure 3B). Interestingly, a similar result has been previously reported in the loss-of-function of Grid2 mice [12],[35]. To rule out potential neuromuscular abnormalities as the cause of this phenotype, a hanging wire test [39] was performed on *Bcl-w^−/−^* mice. No obvious differences in the retention time between *Bcl-w^−/−^* and wild type mice were observed (data not shown**)**, suggesting that the lack of motor learning evident in *Bcl-w^−/−^* mice in the Rotarod assay is due to defects in cerebellar function.

In summary, these results demonstrated that both *Bcl-w^−/−^* and *Grid2^ho−4J(−/−)^* mice had the similar phenotypes including significantly lengthened mitochondria in Purkinje cell dendrites, fewer and malformed Purkinje cell/parallel fiber synapses, and motor learning defects.

### Bcl-w and Grid2 Are Both Required for the Normal Development of Purkinje Cell Dendritic Arbors and Spines

Visually, *Bcl-w^−/−^Grid2^ ho−4J (−/−)^* double mutant mice were immediately distinguishable from wild type and the single mutant animals by the fact that they were smaller in size, moved very little, and when prodded moved with an extremely ataxic gait. These motor difficulties were so severe that these mice could not be properly tested in the Rotarod assay (data not shown).

To examine these double mutant mice for histological abnormalities, the Golgi impregnation technique was used to further visualize the architecture of Purkinje cell dendrites and the morphology of dendritic spines (Figure 4A). The Strahler method of ordering (Figure 4B) was subsequently applied to obtain quantitative estimates on the impact of these mutations on the complexity of dendritic trees [40],[41]. In the Strahler method of ordering, each dendritic arbor is assigned as “order” such as primary, secondary, tertiary, etc. Dendritic arbors in each order are then subsequently quantified (Figure 4B). An analysis of the data using the Strahler method demonstrated that the single mutant mice did not have significant differences in each of six Strahler orders compared with that of wild type mice. By contrast in the double knockout mice, Purkinje cell dendritic arbors were reduced significantly that there were four or five Strahler orders compared to six orders in wild type and single knockout mice. Furthermore, the number of branches in each order was also reduced significantly in the double mutant mice in comparison to the single knockout and wild type mice (Figure 4B; Table 2).

**Table 2:** Purkinje Cell Dendritic Defects in *Bcl-w^−/−^Grid2^ho−4J(−/−)^* Mice.

Genotypes	Number of Purkinje Cell Branches in Each Strahler Order						
Wild Type	I	II	III	IV	V	VI	
Cell 1	319	84	21	5	2	1	
Cell 2	201	56	15	5	2	1	
Cell 3	289	81	21	5	2	1	
Cell 4	221	65	20	7	2	1	
Mean±S.E.M	257.5±27.8	71.5±6.3	19.3±1.4	5.5±0.5	2	1	
***Bcl-w^−/−^***							
Cell 1	199	56	21	6	2	1	
Cell 2	192	59	17	5	2	1	
Cell 3	230	66	20	6	2	1	
Cell 4	238	64	21	7	2	1	
Mean±S.E.M	†214.8±11.3	*61.3±2.3	19.8±0.9	‡6.0±0.4	2	1	
***Grid2^ho−4J(−/−)^***							
Cell 1	321	85	24	5	2	1	
Cell 2	313	94	26	5	2	0	
Cell 3	250	66	17	4	2	1	
Cell 4	271	67	21	5	2	0	
Mean±S.E.M	†288.8±16.9	*78±6.9	22±2.0	‡4.8±0.3	2	0.5	
***Bcl-w^−/−^Grid2^ho−4J(−/−)^***							
Cell 1	60	13	3	1	0		0
Cell 2	97	25	6	2	1		0
Cell 3	88	26	6	1	0		0
Cell 4	99	27	6	3	1		0
Cell 5	64	22	5	1	0		0
Cell 6	107	27	7	2	1		0
Mean±S.E.M	85.8±7.9	23.3±2.2	5.5±0.6	1.7±0.3	0.5±0.2		0

Purkinje cell dendritic abnormalities in *Bcl-w^−/−^Grid2^ ho−4J(−/−)^* mice were quantified using the Strahler system of topological analysis on Golgi-stained Purkinje cells. The number of the dendritic branches in each Strahler order was counted for several Purkinje cells for wild type, single knockout, and double knockout mice. Statistic analysis was performed between two genotypes of mice in each order using student t-test. Purkinje cells in *Bcl-w^−/−^Grid2^ho−4J(−/−)^* mice are significantly different from all other genotypes in every Strahler order (p<0.01). ^†^, ^*^, ^‡^ Indicate the significant difference (p<0.05) in the first, the second, and the fourth Strahler order between *Bcl-w^−/−^* and *Grid2^ho−4J(−/−)^* mice.

Additionally, we also found that ∼45% Purkinje cells analyzed in the double mutant mice contained two dendritic branches rather than the single primary branch that extended from the Purkinje cell soma. This compared to ∼5% Purkinje cells analyzed in wild type and the single mutant animals (Table 3).

**Table 3:** Purkinje Cell Primary Dendrite Defect in *Bcl-w^−/−^Grid2^ho−4J(−/−)^* Mice.

Genotypes	Wild type	*Bcl-w^−/−^*	*Grid2^ho−4J(−/−)^*	*Bcl-w^−/−^Grid2^−ho−4J(−/−)^*
	(40 cells)	(40 cells)	(40 cells)	(47 cells)
Percentage of Purkinje Cells With Two Branches Extended From Cell Body	5%	5%	5%	44.9%

Quantification of the fraction of Purkinje cells with two branches issuing from the cell soma is presented.

**Figure 5:**
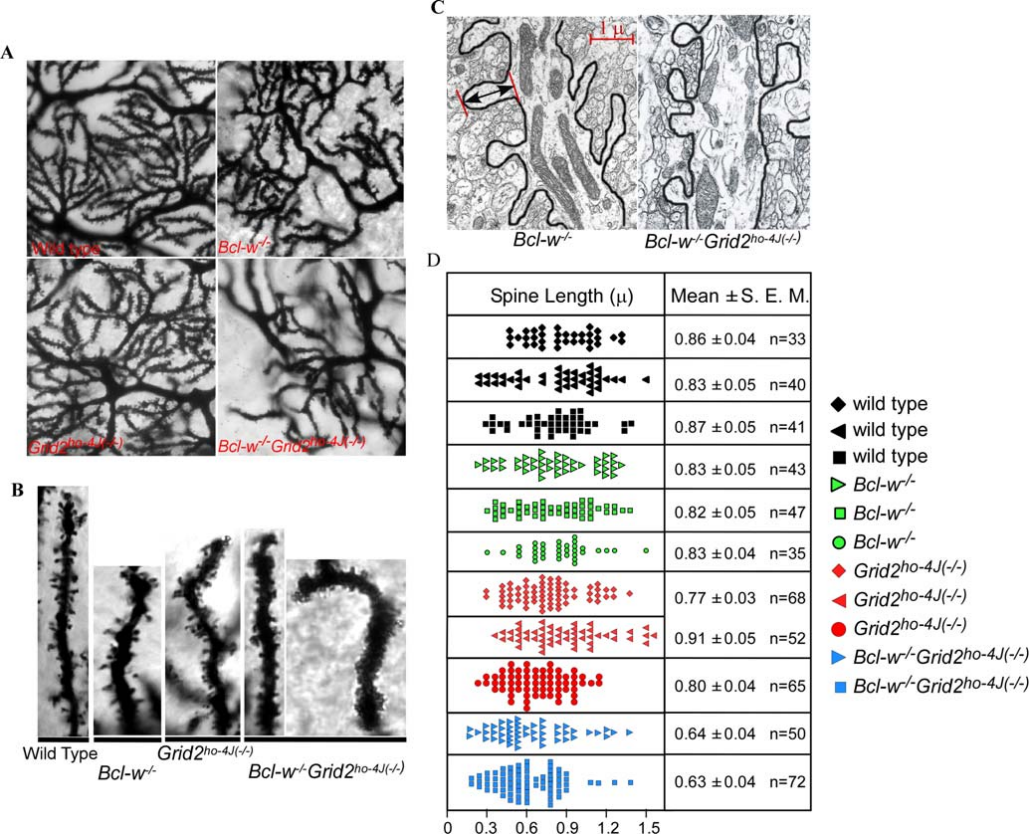
Golgi Stains Illustrating Dendritic Spine Abnormalities in *Bcl-w^−/−^Grid2^ ho−4J(−/−)^* Double Mutant Mice. (A) Golgi stain showing Purkinje dendritic branches and spines in wild type, *Bcl-w^−/−^*, *Grid2^ ho−4J(−/−)^*, and *Bcl-w^−/−^Grid2^ ho−4J(−/−)^* mice. Note the difficulty in visualizing dendritic spines decorating the dendrites of *Bcl-w^−/−^Grid2^ ho−4J(−/−)^* mice. (B) High resolution photographs of terminal dendritic branches as visualized using Golgi stain indicate shorter and more crowded spines in *Bcl-w^−/−^Grid2^ ho−4J(−/−)^* mice. (C) Electron micrograph illustrating the profiles of dendritic spines in the wild type (left panel) and the *Bcl-w^−/−^Grid^ ho−4J(−/−)^* Purkinje cells (right panel). Only those spines that connected to the dendrites and whose profiles clearly delineated both the head and the neck were measured. Note the stubby appearance of the spines present on the double mutant Purkinje cells. Arrow and bars demonstrate the measurement of the spine length. (D) Histogram demonstrating the distribution of spine lengths based on electron micrographic measurements for each genotype. Each color represents one genotype, and each lane one mouse. Student t-test statistics was applied to calculate significant differences in spine length between all pairs of samples. The spine lengths in *Bcl-w^−/−^Grid2^ho−4J(−/−)^* mice were significantly different from those measured in all other genotype samples, p≤0.001, and there were no significant difference among spine lengths from wild type and single mutant mice.

Examination of Purkinje cell dendritic spines using Golgi impregnation in 4–6 mice (Figure 5A–D) for each genotype revealed that Purkinje cell dendritic spines in wild type, *Bcl-w^−/−^*, and *Grid2^ho−4J(−/−)^* mice all had the characteristic door knob-shaped structure and spacing expected, whiles in *Bcl-w^−/−^Grid^ ho−4J(−/−)^* mice they were crowded onto dendrites, appeared significantly shorter, and often branched or lacked a clearly distinguishable spine head or neck (Figure 5A, B). Similar spine defects were found in EM studies in an additional three double mutant mice.

To quantify the difference in spine length between single and double mutant mice, we measured spine profiles that have their necks connected to dendrites (Figure 5C). The wild type Purkinje cell spine lengths determined by this method (0.86±0.33 μ; Figure 5D) were agreed very well with previous measurements of mouse Purkinje cell dendritic spine length (0.87±0.21 μ) obtained using confocal microscopy of Lucifer yellow injected Purkinje cells [42]. In this study, wild type and single mutant mice had similar length of spines, although the spine length decreased significantly by ∼25% in *Bcl-w^−/−^Grid2^ ho−4J (−/−)^* Purkinje cells (Figure 5C, D).

In summary, these results demonstrated that normal Purkinje cell dendrite development and synapse formation require both Bcl-w and Grid2. Since both dendritic arbor number and spine length in double mutant mice are much more severely affected than the sum of these defects in single mutant mice, we conclude that the interaction between Bcl-w and Grid2 genes in regulation of dendrite and spine development is synergistic.

### No Changes in Autophagy Are Evident in Purkinje Cells of *Bcl-w^−/−^*, *Grid2^ho−4J(−/−)^*, or *Bcl-w^−/−^Grid2^ho−4J(−/−)^* Mice

Two recent reports demonstrated that a basal level of autophagy was required for preventing the accumulation of protein aggregates and inclusion bodies and the survival of Purkinje cells [22],[23]. Since Bcl-w can interact physically with Beclin1 and inhibit starvation-induced autophagy (unpublished results), and the dominant Grid2^Lc^ mutation can induce autophagy in Purkinje cells, it is possible that *Bcl-w^−/−^* and *Grid2^ho−4J(−/−)^* mutations might potentially alter autophagy in Purkinje cells and result in observed phenotypes in these cells. We thus examined anatomic evidences of autophay in Purkinje cell EM sections of single and double mutant mice.

It has been shown previously that morphologic evidence for the activation of autophagy indicated by the presence of autophagosomes was readily apparent in Grid2^Lc^ Purkinje cells [20]. In contrast, inhibition of basal autophagy in Purkinje cells can result in the accumulation of inclusion bodies or protein aggregates [22],[23]. However, in a careful examination of the Purkinje cell cytoplasm in wild type and single and double mutant animals in electron micrograph, no morphological evidence indicative of alterations in autophagy was observed (data not shown), suggesting that autophagy is unlikely to be the cause of Purkinje cell phenotypes in mutant mice.

## Discussion

### Bcl-w Regulates Mitochondrial Length in Purkinje Cell Dendrites through Promoting Mitochondrial Fission

During mitochondrial fission process, the mitochondrial fission protein complexes localize on the fission sites, which appear as constriction sites during ongoing mitochondrial proliferation [2],[3]. When the mitochondrial fission is blocked, the constriction sites persist and can be identified easily under the electron microscope [43]. Both the growth of mitochondria and the mitochondrial fission/fusion processes determine the final size or length of the mitochondria in cells. However, it should be notified that mitochondrial growth alone does not generate constriction sites. The frequently observed constriction sites in mitochondria of double mutant mice strongly support that the lengthened mitochondria in single and double mutant mice are due to the slowdown of mitochondrial fission process. Since small mitochondria are seemingly depleted in dendrites that contain extremely long mitochondria in the semi-thin section of double mutant mice, this also supports that slow mitochondrial fission led to the decreased number of mitochondria. However, we cannot rule out that these mitochondrial phenotypes were due to enhanced fusion process.

The survival members of the Bcl-2 family have not been previously reported to regulate mitochondrial length or mitochondrial fission/ fusion in mammalian cells. However, the pro-death members of Bcl-2 family, Bax and Bak have been demonstrated to regulate mitochondrial fission and fusion in both apoptotic and living healthy cells [43]–[45]. For example, in *C. elegans*, overexpression of EGL-1 can induce mitochondrial fission and apoptosis [46]. In mammals, Bax or/and Bak promote mitochondrial fission in apoptotic cells through regulating mitochondrial fission proteins directly [43] or indirectly [47]. In these cells, Bax could also inhibit mitochondrial fusion [44]. However, in living cells Bax and Bak act oppositely as they function to promote mitochondrial fusion [45]. We show in this study that the survival member, Bcl-w has similar characteristics. Thus, Bcl-w promotes mitochondrial fission in Purkinje cells, whiles in testis it protects cells that should normally die in apoptosis.

### Grid2 Regulates Mitochondrial Length in a Distinct Pathway From Bcl-w

The excitatory receptors have not been previously reported to regulate mitochondrial length or dynamics. Previous studies though have demonstrated in neuronal culture system that synaptic activity can stimulate mitochondrial fission and clustering to the dendritic spines [7]. In vivo results for this regulation are lacking, however. Our results implicate that the excitatory receptor Grid2 regulates mitochondrial morphology in addition to its previously found regulation of channel activity and other functions.

The in vivo analysis of mitochondrial localization in Purkinje cells in the current study [37] indicates that mitochondria are normally present in dendrites, but rarely inside dendritic spines. Thus, the actions of Grid2 from synapses on mitochondria may be indirect because Grid2 is localized in Purkinje cell/parallel fiber synapses. The synergistic effect in Bcl-w and Grid2 double mutant mice on mitochondrial length rules out the possibility that Grid2 promotes mitochondrial fission mainly through regulating Bcl-w, and suggests that other pathways might be responsible for Grid2 in regulation of mitochondrial length or fission/fusion. Our studies in the dominant Grid2^Lc^ mutation demonstrated the extensive mitochondrial fragmentation in cytoplasm of Purkinje cell of Lurcher mice during the postnatal development (unpublished results). This suggests that Grid2 might control mitochondrial length through the mitochondrial fission process by regulating calcium influx. Indeed, calcium has been shown in several studies to stimulate mitochondrial fission by regulating the activities of dynamin and the dynamin-like large GTPase, Drp-1 [6]. Thus, Grid2 is likely to function to promote mitochondrial fission through its channel activity.

### Mitochondrial Morphogenesis in Purkinje Cell Dendrites Might Be Important for Synapse Formation and Motor Learning

In this study, we observed fewer Purkinje cells/parallel fiber synapses, an increased ratio of naked spines, and motor learning defect in the double mutant mice. This may be correlated with slowdown of mitochondrial fission in *Bcl-w^−/−^* and *Grid2^ ho−4J(−/−)^* mice, as suggested by our studies. A more direct correlation between mitochondrial fission or fusion and the number of spines and synapses has been demonstrated in primary neuronal culture; overexpression of mitochondrial fission protein Drp-1 in these cells resulted in increased number of mitochondria, correlated with increased number of spine and synapse. In contrast, the expression of mitochondrial fusion protein, OPA1 or dominant negative version of Drp-1 has been reported to lead to fewer numbers of spines and synapses [7]. These results implicate that mitochondrial fission in healthy cells might serve as a means to increase the number of mitochondria to meet energy demands during neuronal growth, or neuronal plasticity, and is likely to be different in mechanism from the excessive mitochondrial fission observed during apoptosis and neurodegeneration. Indeed, the mitochondrial fission during apoptosis can result in the loss of mitochondrial DNAs and lower the function of mitochondria [6]. However, in living healthy human cells, Benard et al. demonstrated recently that when mitochondrial fragmentation was inhibited, a strong inhibition of mitochondrial energy production was observed [48].

Bcl-w is localized in Purkinje cell dendrites and acts on mitochondria, the synaptic defects in *Bcl-w^−/−^* mice are thus likely to be the consequence, not the cause of the mitochondrial morphogenesis defects. Since mitochondrial fission resulted from the neuronal excitation is linked to the danger of degeneration, it is intriguing that the survival member, Bcl-w could promote mitochondrial fission, and has protective function to cells as well.

In summary, the results in the current study suggest that the Bcl-2 family member, Bcl-w, and the excitatory receptor Grid2 can regulate the mitochondrial fission and thus mitochondrial length in dendrites. Altered mitochondrial length in mutant mice of these genes in turn results in abnormalities in synapse formation in the mice.

### Bcl-w and Grid2 Are Both Required for the Development of Purkinje Cell Dendrite and Spines

The Bcl-2 family has not been shown previously to regulate neuronal dendrite development; its effect on neuronal growth has been only associated with cell death. In comparison, the NMDA receptor, one of the glutamate receptor family members has been demonstrated to regulate the activity-dependent dendrite development [49]. However, it is not known if this function of the NMDA receptor has anything to do with mitochondrial morphogenesis or Bcl-2 family members. In this study, we demonstrate that the normal development of Purkinje cell dendrite, dendritic spine, and synapse formation requires both Bcl-w and Grid2, and their regulation of mitochondrial morphogenesis.

Mitochondrial proliferation is a biological process that is associated with cellular division and growth. It takes normally three weeks for Purkinje cells to grow from cell bodies into fully-grown trees with extensive synaptic connections [50]. During this period of time, mitochondrial number also increases significantly. This mitochondrial growing process cannot be exhibited well in cultured Purkinje cells that contain very short and little branched dendritic arbors, unfortunately [51]. The significantly inhibition of mitochondrial fission can thus result in decreased small mitochondrial number and the large size of fused mitochondria that result in reduced mitochondrial motility in Purkinje cell dendrites. These could place an intrinsic limitation on the local energy production and calcium buffering in dendrites, resulting in a failure of perhaps neuronal development and function such as the dynamic growth and branching of dendrites, the development and plasticity of dendritic spines and synapses, channel activities, and the formation of the postsynaptic density, thus leading to the severe morphological defects observed in the double mutant Purkinje cells.

The abundance of mitochondrial fission during the Purkinje cell growth is also balanced or controlled by mitochondrial fusion. A recent paper demonstrated that the absence of mitochondrial fusion protein Mfn2 during Purkinje cell development resulted in excessive mitochondrial fragmentation and Purkinje cell degeneration, suggesting that mitochondrial fusion is required to prevent cells from degeneration [51]. Similarly, the reason that we did not observe any Purkinje cell death in *Bcl-w^−/−^Grid2^ ho−4J(−/−)^* mice in spite of the extensive loss of dendrites, spines, and synapses is likely due to the protective effect by extensively fused mitochondria in these cells. Mitochondrial fusion has also been shown in cultured cells to protect cells from cell death [52],[53].

The early developmental defects in Purkinje cell primary branches in *Bcl-w^−/−^Grid2^ ho−4J(−/−)^* mice indicate that dendritic defects, at least initially, are caused intrinsically, not due to the granule cell parallel fiber innervations because these innervations occur later than the emergence of Purkinje cell primary branches [50].

### Future Prospects

It has been hypothesized that mitochondrial respiration and metabolism may be spatially and temporally regulated by mitochondrial morphology and location that can be integrated to multiple pathways of cellular function [54]. The regulation of mitochondrial length that can result from mitochondrial fission or fusion thus might participate in other pathways that control dendrite and spine morphology and synapse formation, such as development, diseases, and in response to many extrinsic factors such as neuronal activity, hormones [55],[56], and chronic stress [57]. Since we have only observed the mitochondrial morphology changes in fixed tissues, we hope that we can also demonstrate that Bcl-w and Grid2 can affect the mitochondrial fission or fusion in a real time system. This system can also be used to understand the mechanism for Bcl-w and Grid2 and their family members to regulate mitochondrial morphology or the mitochondrial fission and fusion cycle. The studies on mitochondrial fission or fusion will yield important knowledge for our understanding of the development and function of central nervous system.

## Methods

### Mouse Strains and Genetic Crosses

*Bcl-w* knockout mice were obtained from Dr. Grant Macgregor’s lab from Emory University (currently at University of California, Irvine). The generation and typing of these mice were described previously [30]. DBA/2J-*Grid2^ho−4J(−/−)^* mice were purchased from Jackson lab. These mice carry spontaneous null mutation of the Grid2 gene with exons 5–8 deleted, resulting in a 170 amino acid loss in the N-terminal LIVBP-like domain [14],[35],[36]. To obtain *Bcl-w^−/−^Grid2^ho−4J(−/−)^* double knockout mice, the male *Grid2^ho−4J(−/−)^* mice were crossed into the female *Bcl-w^−/−^* mice to obtain *Bcl-w* ^+/−^ *Grid2^ho−4J(+/−)^* mice in F1 generation. Both male and female *Bcl-w^+/−^Grid2^ho−4J(^* ^+/−*)*^ mice from F1 generation were selected and crossed with each other to obtain F2 generation mice. Pups in F2 generation demonstrating the “hotfoot” ataxic phenotypes were identified as homozygous Grid2. Molecular genotyping [30] was applied to distinguish *Grid2^ho−4J(−/−)^*, *Bcl-w* ^+/−^ *Grid2^ho−4J(−/−)^*, and *Bcl-w* ^−/−^ *Grid2^ho−4J(−/−)^* animals. *Bcl-w^−/−^Grid2^ho−4J(+/−)^* mice do not have an obvious ataxia phenotype because mice obtained from the cross using *Bcl-w^+/−^Grid2^ho−4J(+/−)^* male and *Bcl-w^−/−^* female mice did not show the obvious ataxic phenotypes.

### Semi-Thin Sections, Electronmicroscopy, and Observation of Mitochondria and Synapses in Cerebellum and Cultured Cells

Mice were perfused with 2.5% glutaraldehyde, and cerebella were sliced sagitally, and each slice was then diced into pieces containing 2–3 folia. The tissue pieces were post-fixed in 1% osmium, treated with 0.5% aqueous uranyl acetate, and then dehydrated through graded alcohol (70, 95, and 100%). After the treatment with propylene oxide, tissue pieces were embedded in a manner allowing sectioning in the sagital plane in Ducupan (Fluka). The blocks were cured in a 60°C degree oven for 2–3 days.

Blocks were cut with a glass knife to get Semi-thin sections of 0.5 micron. The sections were then stained with 0.25% toluidine blue in 1% sodium borate, and evaluated at the light microscope (LM) level to select the tissue orientation of sagital and longitudinal sections. Photographs of mitochondria were taken in the molecular layer approximately 1/3 of the distance between the pia and Purkinje cell monolayer using a 100X oil lens in a Zeiss Axioplan light microscope and MetaVue acquisition software (Universal Imaging). Four wild type, four *Bcl-w^−/−^*, three *Grid2^ho−4J(−/−)^*, and three *Bcl-w^−/−^Grid2^ho−4J(−/−)^* were examined.

To obtain the EM pictures, the sagital and longitudinal block faces were trimmed, and ultra-thin silver sections were cut with a Reichert-Jung Ultracut E ultramicrotome with a Dupont diamond knife, and collected on copper grids, stained with saturated aqueous uranyl acetate, and lead citrate before examination in Jeol 100 cx electron microscope operated at 80 Kv. EM photographs were taken on dendritic tracks randomly in the molecular layer approximately 1/3 of the distance between the pia and Purkinje cell monolayer in sections collected from several different blocks. Mitochondria profiles were traced and measured from the longitudinal dendritic tracks of each set of photographs.

For morphometric analysis of synapses, only one section was collected on each grid. After establishing the orientation, locating the pia and Purkinje cell layer, images were recorded from regions 1/3 down from the pia at primary magnification of 6,600X x. The print magnification was 16,500X. For locating proximal spines on the Purkinje cell primary branches, the block face was reduced allowing more sections to be collected per grid.

### Accelerating Rotarad and Hanging Wire

The male wild type mice were crossed to the female *Bcl-w^−/−^* mice to obtain *Bcl-w^+/−^* mice, which were subsequently inbred with each other to obtain littermates of wild type, *Bcl-w^+/−^*, and *Bcl-w^−/−^* mice. Six to eight weeks old of these littermates were tested on accelerating Rotarod with 0.1 round/second as starting speed and 0.4 round/second^2^ as accelerating speed. Retention time were begin to be recorded when the mice were placed on the rotating bar and acceleration was applied, and stopped when they failed to run on the rotating bar. Each mouse was given three trials per day for five constitutive days.

The hanging wire test was performed by placing mouse on the top of a wire cage lid, and after mouse grip the wires, lid was turned upside down [39]. The retention time for the mouse to hold wire was recorded. Four *Bcl-w^−/−^* and wild type mice were tested, respectively.

### Paraffin Sectioning and Counting of Purkinje Cells

Mice were intracardially perfused with 4% paraformaldehyde in PBS, and their brains were subsequently dissected and post-fixed overnight. Brains were then dehydrated with 70, 95, and 100% ethanol, and treated in organic solvent, butanol for three days before replacing it with paraffin. 10 μ sections were obtained from paraffin-embedded brain. These sections were then treated with xylene to remove wax and rehydrated before staining them in Cresyl violet and being mounted on slides. Sections obtained in the region close to midline were selected and counted for Purkinje cells on all folia.

### Golgi Stain, Imaging of Purkinje Dendrites, and Spines

Golgi stain of mouse brain was obtained using FD Rapid GolgiStain Kit (FD NeuroTechnologies, Inc.) according to the manufacture’s instruction. The images of Purkinje cells were collected using the DIC microscope (Zeiss) with 10X object lens (Figure 4A, upper panel) and with 5X lens (Figure 4A, lower panel). To analyze Purkinje dendrite branches using the Strahler method of ordering, a Z-stack of Purkinje cell images was collected using MetaVue acquisition software (Universal Imaging), and 20X water lens, and used to quantify Purkinje cell dendritic branches. The spines and dendrites were photographed from combined images from Z-stack (Figure 5A). The spines on dendritic branches of the first Strahler order (Figure 5B) were photographed using 100X oil lens. The number of mice examined was indicated in Table 1.
